# Type 1 diabetes defined by severe insulin deficiency occurs after 30 years of age and is commonly treated as type 2 diabetes

**DOI:** 10.1007/s00125-019-4863-8

**Published:** 2019-04-10

**Authors:** Nicholas J. Thomas, Anita L. Lynam, Anita V. Hill, Michael N. Weedon, Beverley M. Shields, Richard A. Oram, Timothy J. McDonald, Andrew T. Hattersley, Angus G. Jones

**Affiliations:** 1 Institute of Biomedical and Clinical Science, University of Exeter Medical School, Barrack Road, Exeter, EX25DW UK; 2 Department of Diabetes and Endocrinology, Royal Devon and Exeter NHS Foundation Trust, Exeter, UK; 3 Renal Department, Royal Devon and Exeter NHS Foundation Trust, Exeter, UK; 4 Academic Department of Blood Sciences, Royal Devon and Exeter NHS Foundation Trust, Exeter, UK

**Keywords:** Autoantibodies, Classification, C-peptide, Genetic risk score, Type 1 diabetes, Type 2 diabetes

## Abstract

**Aims/hypothesis:**

Late-onset type 1 diabetes can be difficult to identify. Measurement of endogenous insulin secretion using C-peptide provides a gold standard classification of diabetes type in longstanding diabetes that closely relates to treatment requirements. We aimed to determine the prevalence and characteristics of type 1 diabetes defined by severe endogenous insulin deficiency after age 30 and assess whether these individuals are identified and managed as having type 1 diabetes in clinical practice.

**Methods:**

We assessed the characteristics of type 1 diabetes defined by rapid insulin requirement (within 3 years of diagnosis) and severe endogenous insulin deficiency (non-fasting C-peptide <200 pmol/l) in 583 participants with insulin-treated diabetes, diagnosed after age 30, from the Diabetes Alliance for Research in England (DARE) population cohort. We compared characteristics with participants with retained endogenous insulin secretion (>600 pmol/l) and 220 participants with severe insulin deficiency who were diagnosed under age 30.

**Results:**

Twenty-one per cent of participants with insulin-treated diabetes who were diagnosed after age 30 met the study criteria for type 1 diabetes. Of these participants, 38% did not receive insulin at diagnosis, of whom 47% self-reported type 2 diabetes. Rapid insulin requirement was highly predictive of severe endogenous insulin deficiency: 85% required insulin within 1 year of diagnosis, and 47% of all those initially treated without insulin who progressed to insulin treatment within 3 years of diagnosis had severe endogenous insulin deficiency. Participants with late-onset type 1 diabetes defined by development of severe insulin deficiency had similar clinical characteristics to those with young-onset type 1 diabetes. However, those with later onset type 1 diabetes had a modestly lower type 1 diabetes genetic risk score (0.268 vs 0.279; *p* < 0.001 [expected type 2 diabetes population median, 0.231]), a higher islet autoantibody prevalence (GAD-, islet antigen 2 [IA2]- or zinc transporter protein 8 [ZnT8]-positive) of 78% at 13 years vs 62% at 26 years of diabetes duration; (*p* = 0.02), and were less likely to identify as having type 1 diabetes (79% vs 100%; *p* < 0.001) vs those with young-onset disease.

**Conclusions/interpretation:**

Type 1 diabetes diagnosed over 30 years of age, defined by severe insulin deficiency, has similar clinical and biological characteristics to that occurring at younger ages, but is frequently not identified. Clinicians should be aware that patients progressing to insulin within 3 years of diagnosis have a high likelihood of type 1 diabetes, regardless of initial diagnosis.

**Electronic supplementary material:**

The online version of this article (10.1007/s00125-019-4863-8) contains peer-reviewed but unedited supplementary material, which is available to authorised users.

## Introduction

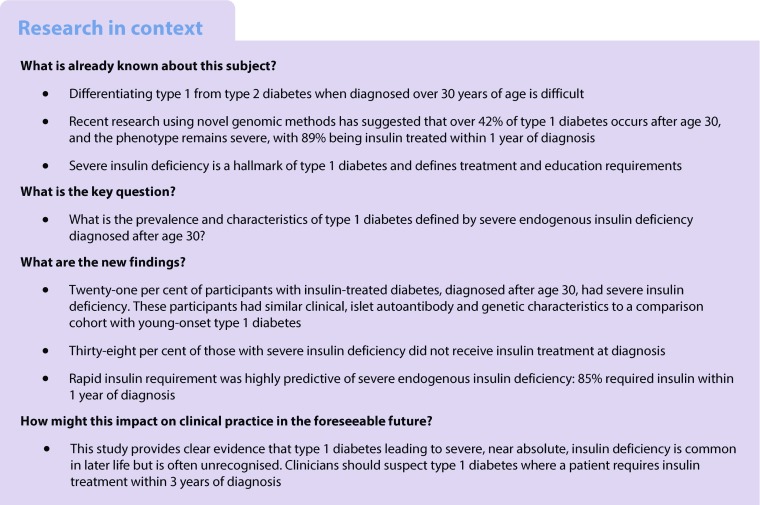



Type 1 diabetes is classically defined by autoimmune or idiopathic beta cell destruction leading to severe insulin deficiency [1], but this aetiopathological definition is difficult to apply in clinical practice. Definitions based on clinical criteria have been little investigated in adults and are poorly defined, with many features commonly thought of as discriminatory, having no evidence base [2]. As a result, there is no robust evidence-based guidance on identifying type 1 diabetes in later life. Recent novel population-based genetic stratification analysis has suggested that at least 42% of type 1 diabetes occurs after the age of 30 [3]. This research suggests late type 1 diabetes has similar characteristics to young-onset disease and, in contrast to type 1 diabetes defined by autoantibody status alone, continues to have a severe phenotype: 89% were treated with insulin within 1 year of diagnosis, and 11% developed ketoacidosis [4]. However, this technique does not allow classification of type 1 diabetes at an individual level, and neither islet autoantibodies nor measures of endogenous insulin secretion were available in the UK Biobank population analysed [3].

Measurement of C-peptide, a surrogate marker of insulin secretion, allows robust diagnosis of type 1 diabetes in long-standing diabetes (>3 years duration) and closely relates to treatment requirements [5]. The development of severe (near absolute) insulin deficiency (commonly defined by stimulated C-peptide <200 pmol/l) results in high glucose variability, marked hypoglycaemia risk, absolute insulin requirement and poor glycaemic response to non-insulin therapies [5, 6]. Therefore, those with severe insulin deficiency will require glycaemic management according to type 1 diabetes guidelines regardless of disease aetiology. The prevalence and characteristics of diabetes leading to severe insulin deficiency in older adults is not known.

We aimed to determine the prevalence and characteristics of type 1 diabetes defined by severe endogenous insulin deficiency in patients >30 years of age with insulin-treated diabetes. We also assessed whether these patients were identified and managed as having type 1 diabetes in clinical practice.

## Methods

We assessed the prevalence and characteristics of type 1 diabetes defined by early insulin requirement and severe endogenous insulin deficiency in an insulin-treated population cohort.

### Participants

583 participants from the population-based Exeter Diabetes Alliance for Research in England (DARE) cohort met the following inclusion criteria: diagnosed with diabetes after 30 years of age, insulin treated and with a C-peptide measurement available. Participants with previous pancreatic pathology (*n* = 2) were excluded from analysis. To allow for comparison with young-onset type 1 diabetes, we assessed a further cohort of 220 DARE participants with age of diabetes diagnosis ≤30 years who met the study criteria for type 1 diabetes (ESM Fig. 1; see below).

The DARE study was approved by the South West ethics committee (UK). Participants gave informed consent.

### Assessment of clinical characteristics

Clinical history was self-reported by participants in an interview with a research nurse, and height and weight were measured at a median diabetes duration of 13 years. Time to insulin was defined as immediate if within 2 weeks of diagnosis.

### Laboratory analysis

Non-fasting (random) C-peptide, islet autoantibodies (GAD, zinc transporter protein 8 [ZnT8], islet antigen 2 [IA2]) and a type 1 diabetes genetic risk score (T1DGRS) were assessed in all included participants, as previously described (see ESM methods) [6, 7]. Where multiple C-peptide measurements were available (70% of participants, median 3 values per participant), the median value was used. Median duration at islet autoantibody and C-peptide assessment was 13 and 16 years respectively (ESM Methods).

### Definition of diabetes type

Type 1 diabetes was defined as continuous insulin treatment commenced within 3 years of diagnosis and severe insulin deficiency defined by a non-fasting C-peptide <200 pmol/l. Insulin-treated type 2 diabetes was defined as current insulin treatment with a C-peptide ≥600 pmol/l and a duration of diabetes of over 3 years at C-peptide measurement. Participants who were insulin treated and with a C-peptide level ≥200–<600 pmol/l (*n* = 115, median 48 months from diagnosis to insulin therapy) were considered indeterminate and were not included in analysis (ESM Fig. 1) [5].

### Statistical analysis

Data were assessed visually for distribution. Data for all continuous variables except age were not normally distributed; therefore, data are presented as median and interquartile range (IQR), unless otherwise stated. We compared the clinical characteristics, islet autoantibody status and T1DGRS of participant groups defined by C-peptide, initial insulin treatment (within 2 weeks of diagnosis) and age at diagnosis using the Wilcoxon rank-sum test for continuous variables and χ^2^ analysis for comparison of categorical characteristics. All analyses were performed using Stata 15 (StataCorp LP, College Station, TX, USA).

## Results

### Severe insulin deficiency occurs in 21% of insulin-treated patients diagnosed after 30 years of age and has similar clinical characteristics to young-onset type 1 diabetes

Twenty-one per cent (123/583) of insulin-treated participants diagnosed with diabetes after 30 years of age met the study criteria for type 1 diabetes and had severe endogenous insulin deficiency (insulin treatment within 3 years and C-peptide <200 pmol/l) (ESM Fig. 1).

The characteristics of participants with late-onset (>30 years of age) type 1 diabetes, young-onset (≤30 years of age) type 1 diabetes and late-onset type 2 diabetes (retained endogenous insulin secretion) are shown in Table 1. Participants with late-onset type 1 diabetes defined by development of severe insulin deficiency had broadly similar characteristics to those with young-onset type 1 diabetes: BMI, insulin dose and HbA_1c_ did not differ. However, those with later onset type 1 diabetes had a modestly lower T1DGRS (0.268 vs 0.279 [expected type 2 diabetes population median 0.231 [8]]), higher islet autoantibody prevalence (78% vs 62%, at 13 vs 26 years duration) and were more likely to be treated as, and identify as having, type 2 diabetes (oral glucose-lowering agent use, 15% vs 5%; insulin at diagnosis, 62% vs 96%; self-reported type 2 diabetes 20% vs 0%).

**Table 1:** Comparison of the characteristics of study participants by diabetes classification and age of diagnosis

Variable	T1D diagnosed >30 (C-peptide <200 pmol/l, time to insulin <3 years)	T2D diagnosed >30 (C-peptide ≥600 pmol/l)	T1D diagnosed ≤30 (C-peptide <200 pmol/l, time to insulin <3 years)	T1D diagnosed >30 vs T2D (*p* value)	T1D diagnosed >30 vs T1D diagnosed ≤30 (*p* value)
Participant (*n*)	123	306	220	–	–
Current age (years)	62 (51–67)	67 (62–73)	45 (33–58)	<0.001	<0.001
Age at diagnosis (years)	44 (36–54)	54 (46–60)	16 (11–22)	<0.001	<0.001
Duration of diabetes at recruitment (years)	13 (6–22)	13 (10–19)	26 (14–40)	>0.1	<0.001
BMI (kg/m^2^)	25.9 (23.0–29.4)	31.6 (28.1–36.2)	26.0 (23.3–28.7)	<0.001	>0.1
Male sex (%)	55 (46, 64)	64 (58, 69)	50 (43, 57)	0.1	>0.1
White ethnicity (%)	99 (96, 100)	96 (93, 98)	95 (91, 97)	>0.1	>0.1
T1DGRS	0.268 (0.242–0.284)	0.229 (0.204–0.249)	0.279 (0.261–0.296)	<0.001	<0.001
T1DGRS >5th centile^a^ of WTCCC reference [12] (%)	82 (74, 88)	45 (40, 51)	96 (92, 98)	<0.001	0.001
C-peptide (pmol/l)	7 (3–65)	1235 (836–1770)	4 (3–13)	<0.001	<0.001
Islet autoantibody positive (%)	78 (67, 87)	6 (3, 11)	62 (53, 69)	<0.001	0.02
Concurrent use of oral glucose-lowering agent (%)	15 (10, 23)	80 (75, 84)	5 (2, 8)	<0.001	0.001
Insulin at diagnosis (%)	62 (53, 70)	10 (7, 14)	96 (92, 98)	<0.001	<0.001
Time to insulin from diagnosis (months)	0 (0–3)	60 (24–120)	0 (0–0)	<0.001	<0.001
Basal bolus insulin regimen/insulin pump (%)	85 (77, 92)	16 (11, 21)	94 (90, 97)	<0.001	0.01
Insulin dose (U/kg)	0.62 (0.46–0.83)	0.50 (0.30–0.86)	0.59 (0.44–0.82)	0.02	>0.1
HbA_1c_ (mmol/mol)	69 (61–82)	62 (56–74)	66 (58–74)	<0.001	0.08
HbA_1c_ (%)	8.5 (7.7–9.7)	7.8 (7.3–8.9)	8.2 (7.5–8.9)	<0.001	0.08
Self-reported T1D (%)	79 (70, 86)	5 (3, 8)	100 (98, 100)	<0.001	<0.001
Self-reported T2D (%)	20 (13, 28)	93 (90, 96)	0 (0, 2)	<0.001	<0.001

Data are presented as % (95% CI) or median (IQR)

^a^Fifth centile of a young type 1 diabetes population corresponding to the 50th centile of a type 2 diabetes population [8]

IQR, interquartile range; T1D, type 1 diabetes; T2D type 2 diabetes; WTCCC, Wellcome Trust Case Control Consortium

### Classical clinical criteria cannot reliably identify individuals with late-onset type 1 diabetes leading to severe insulin deficiency

Despite similar clinical features to young-onset type 1 diabetes, classical clinical criteria could not robustly identify late-onset type 1 diabetes. Only 41% had a BMI <25 and 28% of participants with a BMI <25 had type 2 diabetes. UK National Institute for Health and Care Excellence guidance for type 1 diabetes identification (age of diagnosis <50 or BMI <25 kg/m^2^) identified 81% of type 1 diabetes cases but had very low specificity (41%). The specificity of these criteria would be far lower had non-insulin treated individuals been included in this cohort.

### Thirty-eight per cent of participants with late-onset type 1 diabetes did not receive insulin at diagnosis

Participants meeting criteria for type 1 diabetes after age 30 who did not receive insulin at diagnosis (38%) commenced insulin a median of 12 months from diagnosis, and had similar characteristics to those commencing insulin at diagnosis (ESM Table 1). However, 47% of those who had delayed insulin treatment reported a diagnosis of type 2 diabetes, and 30% received co-treatment with oral glucose-lowering therapy; this is in marked contrast with the 7% who received oral glucose-lowering therapy in the group who received insulin from diagnosis (*p* < 0.01).

### Early progression to insulin is a strong predictor of future severe insulin deficiency

**Fig. 1:**
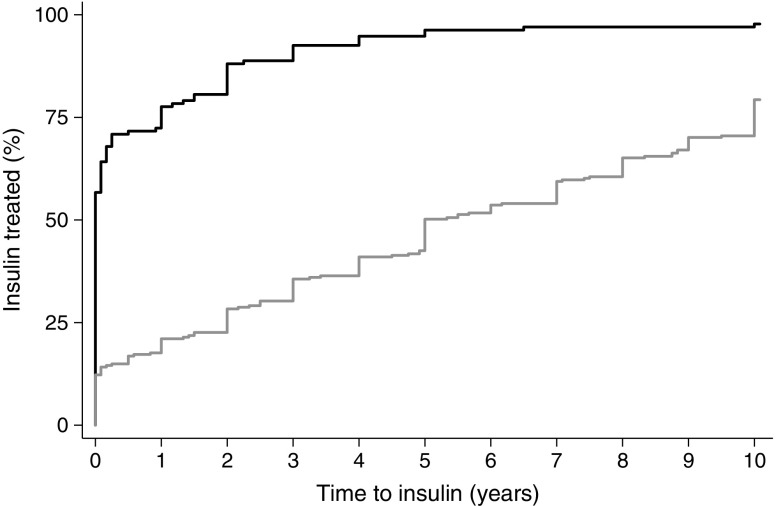
Comparison of time to insulin therapy up to 10 years post diabetes diagnosis in participants with severe endogenous insulin deficiency (C-peptide <200 pmol/l; black line) and participants with retained endogenous insulin secretion (C-peptide ≥600 pmol/l; grey line)

Eighty-five per cent (104/123) of all participants meeting criteria for type 1 diabetes (C-peptide <200 pmol/l and insulin therapy within 3 years) in the cohort diagnosed >30 were treated with insulin within 1 year of diagnosis (78% [104/133] for those defined by low C-peptide only) vs 18% (55/306) of those meeting criteria for type 2 diabetes (Fig. 1). Thirty-two per cent (37/115) of those with an intermediate C-peptide (≥200–<600 pmol/l) required insulin within 1 year of diagnosis. Of all those progressing to insulin within 3 years of diagnosis, 47% (123/264) met the study criteria for type 1 diabetes and 18% (48/264) had intermediate C-peptide (≥200–<600 pmol/l). Severe insulin deficiency was rare in those progressing to insulin after 3 years, occurring in only 10 of 231 participants (4%; Fig. 1).

### Thirty per cent of those diagnosed >30 years of age and administered insulin at diagnosis had type 2 diabetes according to C-peptide criteria

Despite clear clinical, biochemical and genetic characteristics of type 2 diabetes (ESM Table 2), 30% of those meeting study criteria for type 2 diabetes were commenced on insulin at diagnosis. In the group meeting study criteria for type 2 diabetes and treated with insulin at diagnosis, 25% reported a diagnosis of type 1 diabetes, and only 59% received concurrent oral glucose-lowering therapy at a median diabetes duration of 10 years (ESM Table 2).

## Discussion

To our knowledge, this is the first population analysis evaluating the prevalence of type 1 diabetes defined by low endogenous insulin secretion. Our study shows that late-onset type 1 diabetes with severe endogenous insulin deficiency is relatively common (21% of insulin-treated patients) and has very similar characteristics to young-onset type 1 diabetes, but is frequently initially treated as type 2 diabetes. Forty-seven per cent of participants progressing to insulin therapy within 3 years had type 1 diabetes and severe endogenous insulin deficiency, but this was often unrecognised.

Our finding that the clinical features of those with late-onset type 1 diabetes are similar to those with young-onset type 1 diabetes is consistent with recent research using a novel genetic stratification methodology [3]. This showed that 89% of those with genetically defined type 1 diabetes occurring after age 30 required insulin within a year, strikingly similar to the 85% we report using a C-peptide-based definition. This is in contrast to diabetes defined solely by islet autoantibody status, where the phenotype appears intermediate between classical type 1 diabetes and type 2 diabetes in this age group [4].

A limitation of this study is the cross-sectional design within a relatively homogenous, geographically restricted population. Time to insulin and age of diagnosis were self-reported, and participants were assessed a median 13 years after diagnosis, meaning islet autoantibody positivity will be lower than at diagnosis [9]. Negative autoantibody tests in this context, therefore, do not exclude autoimmune diabetes, and we consider it likely that the aetiology of antibody-negative participants with low C-peptide will be autoimmune, as suggested by the high T1DGRS of these participants (median 0.262, data not shown). Islet autoantibodies were measured after a longer duration of diabetes in the young-onset cohort compared with those diagnosed later in life, potentially explaining the higher rate of islet autoantibody positivity observed in the late-onset cohort. A further limitation of this study is the lack of availability of a concurrent glucose level when C-peptide was measured; it is recognised that hypoglycaemia can result in a reduced C-peptide result [10].

These results have clear implications for clinical practice. They show that type 1 diabetes leading to endogenous insulin deficiency is common in later life but is difficult to identify. Consistent with this, many participants with type 1 diabetes in our cohort were diagnosed and treated as having type 2 diabetes. Without a diagnosis of type 1 diabetes, a patient will not receive appropriate education and will not be eligible for interventions that are often restricted to those with type 1 diabetes, such as carbohydrate counting, continuous glucose monitoring and insulin-pump therapy. They will be at risk of ketoacidosis if insulin is withdrawn. Our results suggest that if patients are treated as having type 2 diabetes but progress to insulin within 3 years of diagnosis, clinicians should reassess the underlying diagnosis and strongly consider biomarker testing [5, 8, 11].

## Conclusion

Type 1 diabetes defined by severe insulin deficiency has similar clinical and biological characteristics to type 1 diabetes occurring at younger ages. However, in later life, patients with type 1 diabetes leading to endogenous insulin deficiency are frequently diagnosed and treated as having type 2 diabetes. Clinicians should be aware that patients progressing to insulin within 3 years of diabetes diagnosis have a high likelihood of having type 1 diabetes, regardless of initial diagnosis.

## Electronic supplementary material

ESM (PDF 180 kb)

## Data Availability

The datasets analysed during the current study are available through application to the Peninsula Research Bank, which is managed by the NIHR Exeter Clinical Research Facility. Information on application or data are available on http://exeter.crf.nihr.ac.uk/content/tissue-banks

## Abbreviations

DARE: Diabetes Alliance for Research in England
T1DGRS: Type 1 diabetes genetic risk score
