# Supplementary material for: Type 1 diabetes defined by severe insulin deficiency occurs after 30 years of age and is commonly treated as type 2 diabetes
Source: Diabetologia. 2019 Apr 10;62(7):1167–72. doi: 10.1007/s00125-019-4863-8 (PMC6559997; doi:10.1007/s00125-019-4863-8)
Supplement: Supplementary file 1 — (PDF 180 kb) [file 125_2019_4863_MOESM1_ESM.pdf]

Electronic supplementary material (ESM)

Methods

Laboratory analysis

Analysis of C-peptide and islet autoantibodies (GAD, IA2, ZNT8) was performed by the Academic department of Blood Sciences Department at the Royal Devon and Exeter Hospital.

Assessment of type 1 Diabetes Genetic Risk score. We calculated the type 1 diabetes genetic risk score (T1DGRS), a measure of participants genetic susceptibility to type 1 diabetes, using published variants known to be associated with type 1 diabetes risk as previously described [2]. The HLA haplotype score (HLA DR haplotype from tag variants) was added to the score of the remaining variants each multiplied by the natural log of the odds ratio and finally divided by the number of alleles. To allow comparison to expected results from clearly defined type 2 diabetes and type 1 diabetes populations, T1DGRS results are also presented as centiles derived within the Welcome Trust Case Control Consortium (WTCCC) type 2 diabetes and type 1 diabetes populations [2]. T1DGRS calculation was not performed if genotyping results were missing for either of the two alleles with the greatest weighting (DR3/DR4-DQ8 or HLA_DRB1_15) or if more than two of any other SNPs were missing.

Assessment of Islet autoantibodies. GAD, IA2 and ZnT8 islet antibodies were measured using ELISA assays (RSR Limited, Cardiff, U.K.) on a Dynex DS2 automated ELISA system (Launch Diagnostics, Longfield, U.K.), at median 13 years diabetes duration. Islet antibodies were available in 74% (482/649) of participants and considered positive if ≥ 97.5th centile of 1559 non-diabetic control subjects (GAD ≥ 11 World Health Organization units/mL, IA2 ≥ World Health Organization units/mL, Znt8 age < 30 years ≥ 65 World Health Organization units/mL , ZnT8 age ≥ 30 years ≥10 World Health Organization units/mL [1]..

Assessment of C-peptide. C-peptide was measured on stored non fasting EDTA at DARE recruitment (non-fasting, participants recruited after January 2010) and from July 2014,

with specific separate participant consent on subsequent routine non-fasting EDTA samples sent as part of routine care. Consent to measure C-peptide on routine clinical samples was sought from all new study participants from May 2014 and all existing insulin treated participants from July 2014. Where more than one C-peptide value was available (70% of participants, with a median 3 samples each), the median C-peptide value was used. The median diabetes duration at C-peptide assessment was 16 years. C-peptide was measured using an electrochemiluminescence immunoassay on a Roche Diagnostics E170 analyser (Roche, Mannheim, Germany).

ESM table 1:- Comparison of participants with severe endogenous insulin deficiency (C-peptide<200 pmol/l) commencing insulin at diagnosis and with delayed insulin treatment.

Variable % (95% CI), or median (interquartile range)	Insulin at diagnosis n= 76	Delayed insulin treatment n=47	p value
Current age (years)	60 (49-64)	64 (55-74)	0.002
Age diagnosed (years)	41 (36 -51)	47 (39-56)	0.06
Duration of diabetes at recruitment (years)	11 (5-22)	17 (8-24)	0.07
BMI (kg/m^2)	26.9 (23.1 – 29.5)	25.5 (22.9-28.9)	>0.1
Sex (% male)	59 (47-70)	49 (34-64)	>0.1
T1DGRS	0.268 (0.241-0.285)	0.266 (0.243-0.282)	>0.1
C-peptide (pmol/l)	7 (3-49)	8 (3-133)	>0.1
Islet autoantibody positive (%)	78 (62-89)	72 (53-87)	>0.1
Treated with concurrent Oral hypoglycaemic agent (%)	7 (2-15)	30 (17-45)	0.001
Insulin regimen basal bolus or pump (%)	88 (77-94)	82 (66-92)	>0.1
Insulin dose (units/kg)	0.61 (0.44-0.78)	0.66 (0.49-0.97)	>0.1
HbA1c (mmol/mol), (%)	69 (57-83), 8.5 (7.4-9.7)	67 (63-80), 8.3 (7.9-9.5)	>0.1
Self-reported type 1 diabetes (%)	96 (89-99)	51 (36-66)	<0.001
Self-reported type 2 diabetes (%)	3 (0-9)	47 (32-62)	<0.001

ESM table 2: Characteristics of participants commencing insulin at diagnosis (within 2 weeks) by C-peptide level

**Variable %(95% CI), or median (interquartile range),**	**C-peptide<200 (n= 76)**	**C-peptide≥600 (n=32)**	**p value**
Current age (years)	60 (49-64)	64 (53-74)	0.03
Age diagnosed (years)	41 (36 -51)	50 (39-62)	0.04
Duration of diabetes (years)	11 (5-22)	10 (6-17)	>0.1
BMI (kg/m^2)	26.9 (23.1 – 29.5)	31.1 (27.9 – 37.1)	<0.001
Sex (% male)	59 (47-70)	69 (50-84)	>0.1
T1DGRS	0.268 (0.241-0.285)	0.232 (0.199-0.251)	<0.001
C-peptide (pmol/l)	7 (3-49)	1305 (919-1815)	<0.001
Islet autoantibody positive (%)	78 (62-89)	6 (0-30)	<0.001
Treated with concurrent Oral hypoglycaemic agent (%)	7 (2-15)	59 (41-76)	<0.001
Insulin regimen basal bolus or pump (%)	88 (77-94)	8 (1-27)	<0.001
Insulin regimen basal only (%)	8 (3-17)	58 (37-78)	<0.001
Insulin regimen mixed insulin (%)	5 (1-13)	33 (16-55)	<0.001
Insulin dose (units/kg)	0.61 (0.44-0.78)	0.40 (0.32-0.96)	>0.1
HbA1c (mmol/mol), (%)	69 (57-83), 8.5 (7.4-9.7)	62 (51-74), 7.8(6.8-8.9)	0.06
Self-reported type 1 diabetes (%)	96 (89-99)	25 (11-43)	<0.001
Self-reported type 2 diabetes (%)	3 (0-9)	69 (50-84)	<0.001

ESM figure 1: Flow diagram of classification methodology. Bold two-way arrows show where comparisons are made.

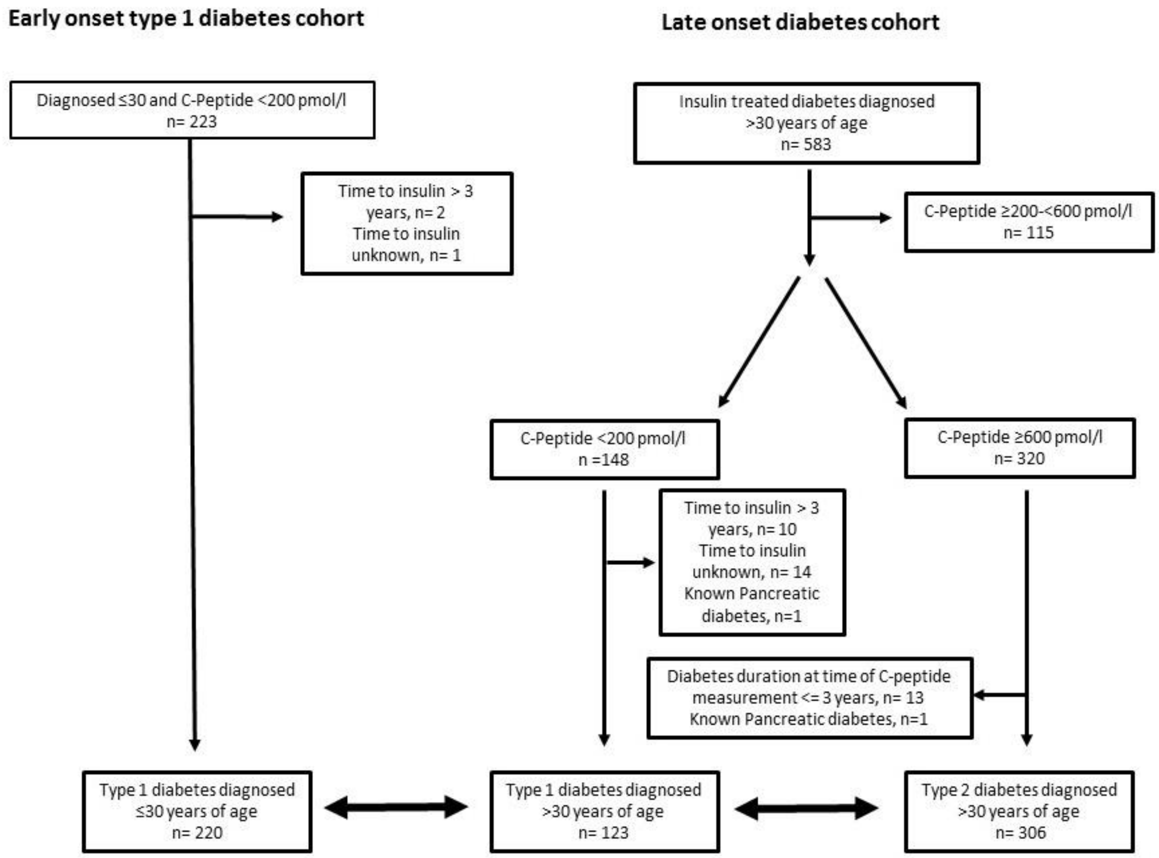

Supplementary references

1. McDonald TJ, Colclough K, Brown R, Shields B, Shepherd M, Bingley P*, et al.* Islet autoantibodies can discriminate maturity-onset diabetes of the young (MODY) from Type 1 diabetes. *Diabetic medicine : a journal of the British Diabetic Association* 2011; **28**:1028-1033.
2. Oram RA, Patel K, Hill A, Shields B, McDonald TJ, Jones A*, et al.* A Type 1 Diabetes Genetic Risk Score Can Aid Discrimination Between Type 1 and Type 2 Diabetes in Young Adults. *Diabetes care* 2015; **39**:337-344.
